# Genetic introgression of ethylene-suppressed transgenic tomatoes with higher-polyamines trait overcomes many unintended effects due to reduced ethylene on the primary metabolome

**DOI:** 10.3389/fpls.2014.00632

**Published:** 2014-12-05

**Authors:** Anatoly P. Sobolev, Anil Neelam, Tahira Fatima, Vijaya Shukla, Avtar K. Handa, Autar K. Mattoo

**Affiliations:** ^1^Laboratory of Magnetic Resonance “Annalaura Segre”, Institute of Chemical Methodologies, National Research CouncilRome, Italy; ^2^Sustainable Agricultural Systems Laboratory, The Henry A. Wallace Agricultural Research Center, Agricultural Research Service, United States Department of AgricultureBeltsville, MD, USA; ^3^Department of Horticulture, Purdue University, West LafayetteIN, USA

**Keywords:** ACC synthase, fruit ripening, *LeACS2*, metabolome, polyamines, spermidine, spermine, transgenics

## Abstract

Ethylene regulates a myriad physiological and biochemical processes in ripening fruits and is accepted as the ripening hormone for the climacteric fruits. However, its effects on metabolome and resulting fruit quality are not yet fully understood, particularly when some of the ripening-associated biochemical changes are independent of ethylene action. We have generated a homozygous transgenic tomato genotype (2AS-AS) that exhibits reduced ethylene production as a result of impaired expression of 1-aminocyclopropane-1-carboxylate synthase 2 gene by its antisense RNA and had a longer shelf life. Double transgenic hybrid (2AS-AS × 579HO) developed through a genetic cross between 2AS-AS and 579HO (Mehta et al., 2002) lines resulted in significantly higher ethylene production than either the WT or 2AS-AS fruit. To determine the effects of reduced ethylene and introgression of higher polyamines’ trait, the metabolic profiles of ripening fruits from WT (556AZ), 2AS-AS, and 2AS-AS × 579HO lines were determined using ^1^H-NMR spectroscopy. The levels of Glu, Asp, AMP, Adenosine, Nucl1, and Nucl2 increased during ripening of the WT fruit. The increases in Glu, Asp, and AMP levels were attenuated in 2AS-AS fruit but recovered in the double hybrid with higher ethylene and polyamine levels. The ripening-associated decreases in Ala, Tyr, Val, Ile, Phe, malate, and myo-inositol levels in the 2AS-AS line were not reversed in the double hybrid line suggesting a developmental/ripening regulated accumulation of these metabolites independent of ethylene. Significant increases in the levels of fumarate, formate, choline, Nucl1, and Nucl2 at most stages of ripening fruit were found in the double transgenic line due to introgression with higher-polyamines trait. Taken together these results show that the ripening-associated metabolic changes are both ethylene dependent and independent, and that the fruit metabolome is under the control of multiple regulators, including ethylene and polyamines.

## INTRODUCTION

Ethylene is a gaseous plant hormone involved in regulating various aspects of plant growth, development, and senescence (Mattoo and Suttle, 1991; Abeles et al., 1992). Among myriad processes that ethylene regulates its role is plant aging, fruit ripening and cell death is pivotal. Plants have evolved to tightly regulate the production of ethylene, which seems to be achieved via the presence of a family of genes encoding key enzymes in ethylene biosynthesis, and by differential regulation of their transcripts (see Fluhr and Mattoo, 1996; Harpaz-Saad et al., 2012; Gapper et al., 2014). Ethylene biosynthesis in higher plants mostly involves the conversion of methionine to *S*-adenosylmethionine (SAM) catalyzed by SAM synthase, SAM is then converted to 1-aminocyclopropane-1-carboxylate (ACC) catalyzed by ACC synthase, and finally, ACC is oxidized to ethylene by ACC oxidase (Harpaz-Saad et al., 2012; Grierson, 2014).

The reverse genetics approach demonstrated that ethylene is indeed a ripening hormone and ACC synthase is a rate-limiting enzyme in the pathway (Oeller et al., 1991). Treatments with chemicals such as inhibitors of ethylene biosynthesis (e.g., aminoethoxyvinylglycine) or perception [e.g., silver salts or 1-methylcyclopropene (1-MCP)] were shown to delay fruit ripening (Mattoo and Suttle, 1991; Abeles et al., 1992). Genetic approach established that silencing ACC synthase or ACC oxidase using the antisense RNA technology leads to delayed ripening and increased shelf life of fruits (Hamilton et al., 1990; Oeller et al., 1991), or by overexpression of a bacterial ACC dreaminess, which hydrolyzes ACC (Klee, 1993). In summary, other genes such as those that encode SAM hydrolase and *N*-ACC malonyltransferase, which catalyze either the synthesis or breakdown of precursors of ethylene biosynthesis and whose expression was manipulated all decreased ethylene (see Fluhr and Mattoo, 1996). Genetic control of ethylene action was also achieved by silencing ethylene receptors and thereby the ethylene signaling transduction pathway (Fluhr and Mattoo, 1996; Wilkinson et al., 1997). However, it is recognized that ethylene-dependent (Gapper et al., 2014), as well as ethylene-independent processes (Hiwasa-Tanase and Ezura, 2014), regulate ripening. In addition to ethylene, other hormones also play a role in the ripening process (Davies and Böttcher, 2014; Hiwasa-Tanase and Ezura, 2014).

Post-harvest losses of fruit and vegetables are significant and therefore stemming such losses has been an on-going activity among horticulturalists and geneticists alike. Early on, increasing the post-harvest life of fruits involved chemical treatment, use of plastic films and treatment with waxes and inhibitors of ethylene biosynthesis or action (Paliyath et al., 2008; Nath et al., 2014). Enhancing the quality attributes particularly nutrients that potentially benefit human and animal health, and flavor/aroma components that add to the produce value has generated considerable research interest among nutraceutical and horticultural industries (Paliyath et al., 2008; Nath et al., 2014).

The association of ethylene with metabolism of sugars and organic acids (Defilippi et al., 2004) as well as aroma volatiles (Bauchot et al., 1998; Flores et al., 2002; Defilippi et al., 2005) brought out an important aspect of fruit physiology. Particularly, such studies highlighted the importance of analyzing nutrients (metabolites) and other quality attributes of fruit and vegetable crops whose shelf-life is extended by treatment with chemical inhibitors of ethylene biosynthesis or by silencing genes that prevent ethylene production. Metabolomics approach has been variously applied to discern the dynamics of metabolic processes in fruits (Carrari et al., 2006; Klie et al., 2013), methyl jasmonate-associated (Kausch et al., 2012) and polyamine-associated metabolic processes (Mattoo et al., 2006) and substrate fluxes into ethylene and polyamine biosynthesis pathways (Lasanajak et al., 2013), as well as to analyze alleles responsible for metabolic traits (Perez-Fons et al., 2014).

Ethylene deficient fruit has been shown to have a prolonged shelf life (Oeller et al., 1991). The question we asked was whether this benefit occurs at the expense of a changed metabolome since ethylene association with metabolic pathways is better appreciated now than previously thought. Therefore, we engineered an ethylene-deficient tomato line by introducing an antisense gene of ACC synthase gene as originally described by Oeller et al. (1991), and also crossed it with a previously described high polyamine tomato line (Mehta et al., 2002; Mattoo et al., 2006) to produce a double transgenic tomato line. A metabolomics approach was then applied to delineate primary metabolites whose levels are affected in the single-event transgenic tomato fruit in comparison to the double transgenic tomato and the azygous control line.

## MATERIALS AND METHODS

### PLANT MATERIAL

The following tomato lines (*Solanum lycopersicum* cv. Ohio 8245) were developed in Ohio 8245 background and analyzed: Azygous control line (556AZ); transgenic 2AS-AS line developed (Methods described below) to anti-sense ACC synthase 2 gene (ACS2) under the constitutive CaMV 35S promoter; and 579X2AS-HO line which is a genetic cross between 2AS-AS and 579HO (see below). Transgenic tomato line 579HO is an independent genotype that expresses yeast SAM decarboxylase (SAMdc) gene under ripening-specific E8 promoter and accumulates polyamines spermidine (SPD) and spermine (SPM), at the cost of putrescine levels, in a fruit ripening-specific manner (Mehta et al., 2002). All the lines were grown in a temperature-controlled greenhouse at the USDA-ARS, Beltsville Agricultural Research Center, Beltsville, MD, USA, under natural lighting. Fruits at the following stages were collected for analysis: Mature green (G), breaker (B), turning (T), pink (P), and red-ripe (R) stages as described previously (Mehta et al., 2002). USDA color chart was used to classify ripening stages

https://ucanr.org/repository/view.cfm?article=83755%20&groupid=9. The pericarp tissue was peeled, weighed, and frozen in liquid nitrogen before lyophilizing (Mattoo et al., 2006).

### DEVELOPING TOMATO PLANTS SUPPRESSED FOR ACC SYNTHASE GENE (2AS-AS LINE) AND ITS SIBLING (2AS-HO × 579HO LINE) - A GENETIC CROSS BETWEEN 2AS-AS AND 579HO TRANSGENIC LINES

**FIGURE 1:**
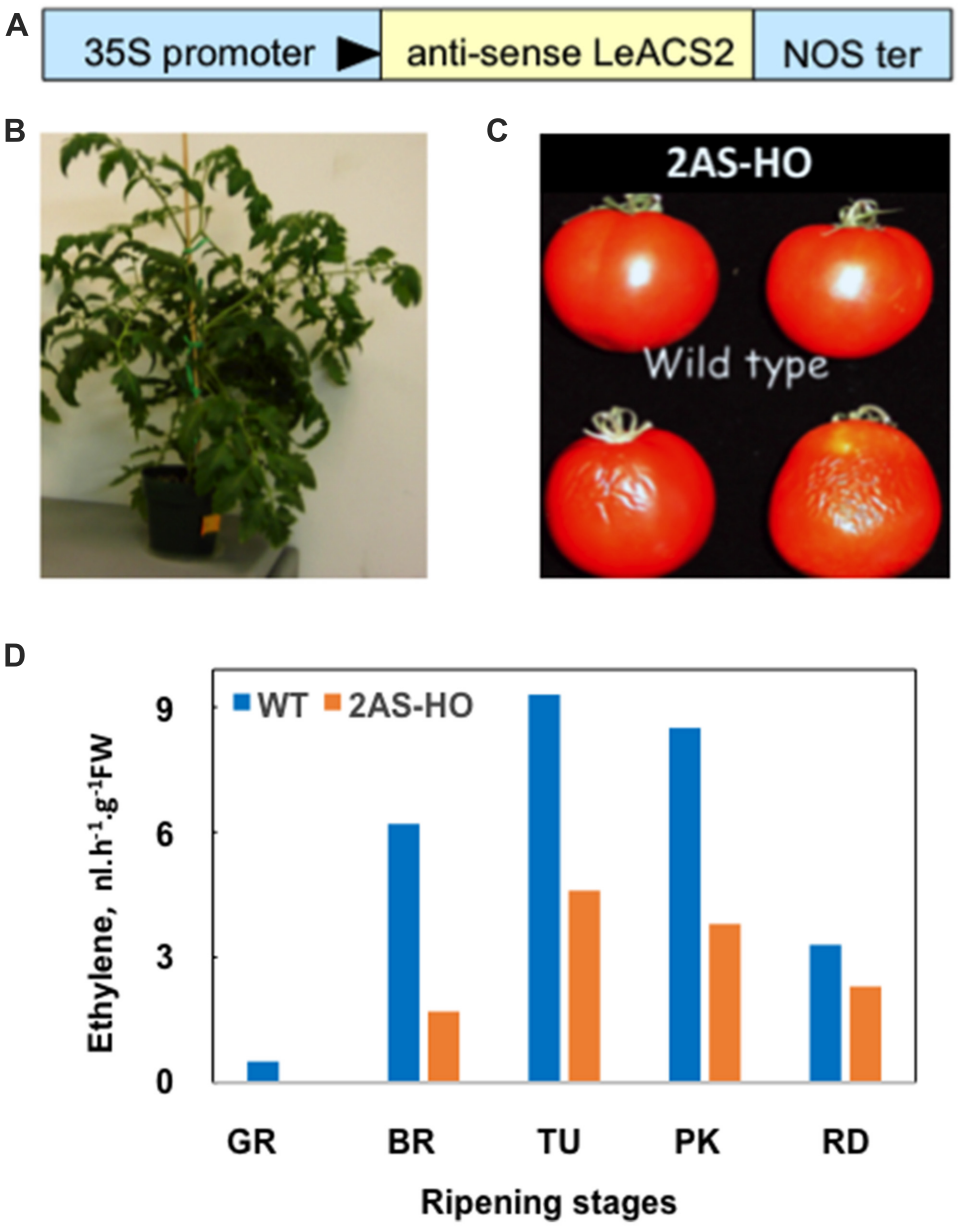
**Generation and characterization of transgenic *SlACS2* antisense tomato. (A)** Anti-sense *SlACS2* construct in pCambia/1303 was made as described in Section “Materials and Methods.” Ohio 8245 tomato cultivar was transformed with *SlACS2*-antisense construct using an *Agrobacterium* based transformation and transgenic plants were selected using hygromycin as selectable marker. **(B)** Phenotype of one of *SlACS2*-antisense tomato plant. **(C)** Phenotype of *SlACS2*-antisense (Upper panel) and WT (Lower panel) fruits taken 45 days of storage at room temperature following green stage. **(D)** Rate of ethylene evolution from fruits of WT and transgenic ACS2-antisense fruits. Each time point represents the average from two fruits. Fruits from *SlACS2* antisense transgenic tomato line produced ∼50% lesser ethylene than the WT line.

Anti-sense *ACS2* transgenic tomato line, 2AS-AS, was developed by *Agrobacterium* mediated transformation. Primers (ACC-SN: 5′AGTGTCGACCCATGGTTAACGAACTAATGGTGAGGG3′ and ACC-XP: 5′GCGTCTAGACACGTGATGGGATTTGAGATTGCAAAGACC3′) with restriction sites (*Nco* I and *Pml* I, respectively) for cloning were designed to span the complete coding region of *LeACS2* gene (Accession #: X59145). *LeACS2* coding region was amplified by PCR with Pfu DNA polymerase (Stratagene) from isolated tomato ACS2 cDNA clone. PCR amplified *LeACS2* was digested with *Pml* I and *Nco* I and cloned into pCambia 1303 binary vector, down stream of a full-length CAMV 35S promoter (CAMBIA, Canberra, ACT, Australia). This resulted in cloning of *LeACS2* coding region in anti-sense orientation with respect to 35S promoter (**Figure 1A**). Binary construct with anti-sense *ACS2* gene was transferred into *Agrobacterium* strain, LBA4404, by electroporation and subsequently used to transform (Pfitzner, 1998) 10-day old cotyledons of tomato (Ohio 8245). Initially, transgenic calli were regenerated and selected on 10 mg/L hygromycin but shoots were regenerated from callus using a lower hygromycin concentration (5 mg/L). Subsequently, the regenerated shoots were rooted on 1 mg/L hygromycin and the plantlets were transferred to soil. The transgenic plants (**Figure 1B**) were screened by PCR using primers (35SST5PF: 5′ AGGACCTAACAGAACTCGCC3′ and ACC-XP: 5′GCGTCTAGACACGTGATGGGATTTGAGATTGCAAAGACC3′) spanning the 35S promoter and anti-sense ACS gene to confirm the presence of promoter and anti-sense ACS transgene. Segregating progeny of T1 and T2 plants containing anti-sense ACS transgene, 2AS-AS, were further selected by PCR.

The selection of 2AS-AS × 579HO heterozygote plants (**Figure 4A**) was made based on the PCR detection of both transgenes (Mehta et al., 2002). Then, F1 plants were selfed and segregating F2 seeds were collected. Evaluation of independent F2 seedlings for the presences of both *ySAMdc* and anti-*SlACS2* transgenes was re-tested by PCR. The selected F2 plants harboring both *ySAMdc* and *SlACS2* transgenes were grown to fruit maturity, and F3 seeds were collected individually from each line. Seeds from each of the independent F3 were germinated and resulting seedling populations evaluated for the transgenes presence as mentioned above. Two lines were obtained in which further segregation of the two transgenes did not occur. One robust one, 2AS-AS × 579HO, homozygous for these transgenes, was selected and grown to maturity. Seed collected from this line were used to grow plants for analysis presented here.

### ANALYSIS OF ETHYLENE

All ethylene measurements were carried out on whole fruits as they attainted indicated ripening stage based on color change (https://ucanr.org/repository/view.cfm?article$=$83755%20&groupid$=$9). Ethylene production in the tomato fruits was measured by gas chromatography with a FID (Mehta et al., 2002).

### NMR ANALYSIS OF FRUIT METABOLITES

Samples for NMR measurement were prepared as previously described (Sobolev et al., 2003; Mattoo et al., 2006). Briefly, dry powder (25 mg) from each sample was rapidly dissolved in 1 mL of 0.4 M sodium phosphate buffer prepared in D_2_O containing known amounts of an authentic standard, 3-(trimethylsilyl)-1-propanesulfonic acid sodium salt (TSP), pH 6.5, and EDTA (0.01 mM). The solution was centrifuged at 10,000 rpm for 7 min and the supernatant filtered to remove any insoluble material. NMR spectra of extracts were recorded at 300 K on a Bruker AVANCE 600 spectrometer operating at the proton frequency of 600.13 MHz. Proton spectra were referenced to the TSP signal (*d* = 0.00 ppm). Proton signals were acquired by co-adding 512 transients with a recycle delay of 3 s. The strong water signal was suppressed by using a NOESY-presat scheme with solvent presaturation during relaxation delay and mixing time (Braun et al., 1998). The one-dimensional spectra were run using 45° flip angle pulses of 6 ms, 32 K data points. After Fourier-transformation and manual phase correction the baseline correction was performed using automatic cubic-spline correction with 25 points distributed over the spectrum. The assignment of ^1^H NMR spectra was performed as previously described (Sobolev et al., 2003; Mattoo et al., 2006; **Table 1**). Additionally, the signals of adenosine, adenosine monophosphate (AMP), tyrosine, histidine, and tryptophan were assigned using literature data (Fan, 1996) and by addition of the corresponding standard compounds (**Table 1**). The singlet at 8.531 ppm was attributed to both adenosine triphosphate (ATP) and adenosine diphosphate (ADP). The intensities of three unassigned signals denoted as B, Nucl1, and Nucl2 were also used in the statistical analysis. The intensity of 30 selected resonances attributed to 30 metabolites (Sobolev et al., 2003; Mattoo et al., 2006) was referenced to the intensity of the internal standard, TSP at 0.00 ppm. The spectra of two to five fruits were analyzed for each ripening stage.

**Table 1:** List of resonances and variables used for the evaluation of the various metabolites.

Variable No.	Chemical shift, ppm	Compound
1	1.020	Ile
2	1.053	Val
3	1.345	Thr
4	1.494	Ala
5	2.085	Glu
6	2.482	Gln
7	2.806	Asp
8	2.908	Asn
9	6.921	Tyr
10	7.249	His
11	7.436	Phe
12	7.748	Trp
13	2.304	GABA
14	2.530	Citrate
15	2.415	Succinate
16	4.316	Malate
17	6.527	Fumarate
18	8.462	Formate
19	3.244	β-Glucose
20	4.023	Fructose
21	5.422	Sucrose
22	3.294	Myo-inositol
23	2.976	^a^B
24	3.208	Choline
25	8.363	Adenosine
26	8.576	AMP
27	8.531	ATP/ADP
28	7.843	^a^Nucl1
29	7.874	^a^Nucl2
30	9.130	Trigonelline

*^a^not assigned*.

### STATISTICAL ANALYSIS

Data of ^1^H-NMR in solution were subjected to statistical analysis by using Statistical software package for Windows (1997; edition by Statsoft) to determine if and to what extent the selected variables were able to distinguish between the different tomatoes and their ripening stages. ANOVA was used to validate the differences. ANOVA and PCA were used to treat the data (Martens and Martens, 2001).

## RESULTS

### ANTI-SENSE LeACS2 TOMATO LINE IS ATTENUATED IN ETHYLENE PRODUCTION AND HAS LONGER SHELF LIFE

Fruits from the wild type (WT) and anti-sense *LeACS2* line, 2AS-AS (line 2.5AS), were found to produce maximum ethylene at the turning stage (Mehta et al., 2002); however, 2AS-AS line produced only 50% of ethylene that produced by the WT (**Figure 1D**). Also, the fruits from ethylene-deficient line had a significantly longer shelf-life as compared to the WT fruits (**Figure 1C**). These data are consistent with established findings in the literature showing that ethylene suppression enhances the shelf life of tomato fruits (Oeller et al., 1991; Paliyath et al., 2008; Nath et al., 2014).

### ALTERED METABOLIC PROFILES IN 2AS-AS LINE VERSUS 556AZ (AZYGOUS CONTROL LINE)

**FIGURE 2:**
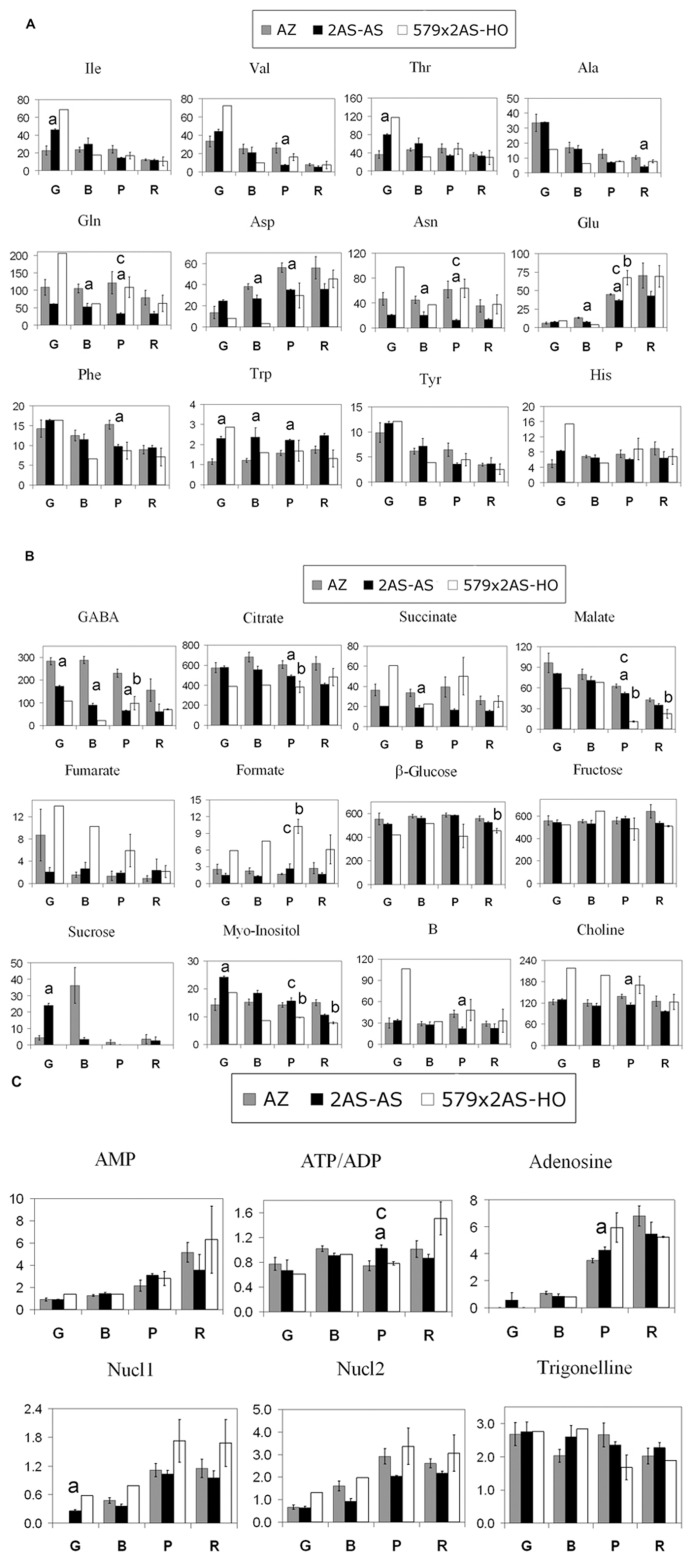
**Molecular abundance of 30 metabolites analyzed in fruit of 556AZ (AZ: gray boxes), 2AS-AS (black boxes), and 579X2AS-HO (white boxes) tomato lines at four ripening stages. (A)** Amino acids. **(B)** GABA, organic acid, and sugars. **(C)** Miscellaneous including heteroaromatic compounds including nucleotides and ATP + ADP. Mean values and SEs are presented. The significant differences (*p*-level < 0.05) between 2AS-AS and 556AZ, 2AS-AS × 579HO (labeled 579 × 2AS-HO) and 556 AZ, 2AS-AS and 2AS-AS × 579HO (labeled 579 × 2AS-HO) are denoted by letters ‘a,’ ‘b,’ and ‘c,’ respectively. G (green), B (breaker), P (pink), and R (red) refer to the stage of the fruit.

Fruits from *2AS-AS* line and 556AZ were harvested from greenhouse-grown plants and prepared as described in the section “Materials and Methods.” Metabolic profiles at four different stages of ripening are shown in **Figures 2A–C**.

### PROFILE OF AMINO ACIDS (LINE 2AS-AS VERSUS 556AZ)

During ripening of fruits from 556AZ control line, the levels of Glu, Asp, His, and Trp gradually increased while Thr level changed slightly, and that of Ile, Val, Gln, Asn, and Phe showed a significant decrease only in R stage (**Figure 2A**). In comparison, the levels of Ile, Val, Thr, Ala, Tyr, Phe, Gln, Asn, and γ-aminobutyrate (GABA) gradually decreased during ripening of fruit from the ethylene-deficient line, 2AS-AS, with the levels of His and Trp remaining constant except for Asp which increased slightly (**Figure 2A**). It is noted that the levels of Gln, Asn, Asp, Glu, and GABA in 2AS-AS line were lower at almost all the ripening stages as compared to the fruits from 556AZ control line, with the exception of Trp that had consistently a higher level in 2AS-AS fruit than the control 556AZ fruit throughout ripening.

### PROFILE OF ORGANIC ACIDS AND SUGARS (LINE 2AS-AS VERSUS 556AZ)

Citrate and malate, the most abundant organic acids in tomato fruit, showed different pattern during ripening: the level of malate gradually decreased in fruit from both 2AS-AS and 556AZ lines, while citrate level was constant in controls and slightly decreased in 2AS-AS line (**Figure 2B**). In contrast to fruit from the 556AZ control line, 2AS-AS ethylene-deficient fruit had lower levels of succinate during ripening and slightly higher level of fumarate except at the green (G) stage (**Figure 2**).

The levels of glucose and fructose were quite stable during ripening without any significant differences between fruit from 2AS-AS and 556AZ lines (**Figure 2B**). Sucrose level was maximal at green (G) stage in 2AS-AS fruit l (about five times higher in comparison with 556AZ G fruits) but, by the breaker (B) stage, the control line (556AZ) fruit had higher levels of sucrose than the 2AS-AS fruit, declining thereafter. Thereafter, the levels decreased dramatically in both genotypes (**Figure 2B**). Myo-inositol level remained constant throughout the ripening of fruit from the control 556AZ line but in the ethylene-deficient 2AS-AS line myo-inositol levels were highest at the green (G) stage and progressively decreased thereafter to being lowest at the red (R) stage (**Figure 2B**).

### PROFILE OF HETEROAROMATIC COMPOUNDS (LINE 2AS-AS VERSUS 556AZ)

The levels of nucleosides and nucleotides also showed a few significant differences between the fruit of ethylene-deficient 2AS-AS line and that of 556AZ control line. Higher levels of adenosine and ATP + ADP at the pink (P) stage and Nucl1 at green (G) stage were observed in the 2AS-AS line in comparison to the fruit from control line (**Figure 2C**).

**FIGURE 3:**
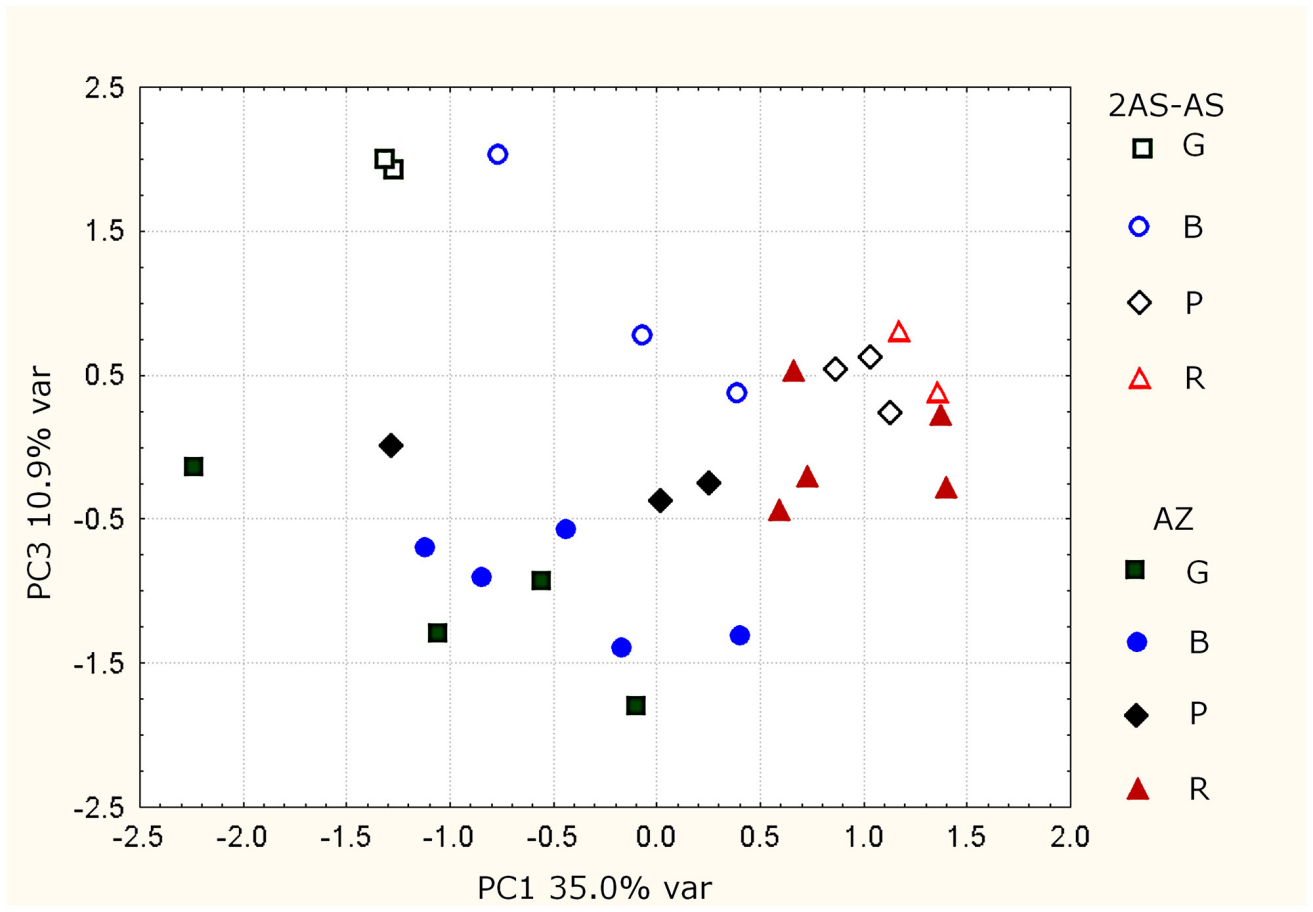
**PCA scores plot of samples from 2AS-AS (open symbols) and 556AZ (AZ, filled symbols) lines.** G (green), B (breaker), P (pink), and R (red) refer to the stage of the fruit.

The multivariate statistical analysis of the data was performed to obtain an overall picture of metabolic differences between ethylene-deficient transgenic fruit and that from 556AZ control line at different ripening stages. First, the Principal Component Analysis (PCA) analysis was applied. The main features of metabolite variability in the fruit from the two lines are visualized in two-dimensional scores plot (**Figure 3**). The obvious distinction in metabolite variability between transgenic (empty symbols) versus control fruits (filled symbols) is observed along PC3 axis. The distribution of samples along PC1 axis reflects the ripeness grade: the lowest and the highest PC1 scores correspond to the green (G) and red (R) fruits, respectively. The separation of samples according to ripeness stage is easily observable for the transgenic line, while the control samples of different ripeness grade are partially mixed. Considering the fraction of variability associated with the principal components (35 % for PC1 and 10.9% of PC3), it is clear that the ripening process is the principal source of metabolite variability, whereas the transgene expression is a secondary factor influencing the metabolic profile. Nonetheless, a good and modest separation according to ripening stage of transgenic and control samples along PC1 suggests a significant influence of transgene expression on ripening. Moreover, the PCA results indicate that the distinction between transgenic and control line is less evident for red tomatoes. The predominant role of ripeness in metabolic changes can mask the influence of transgene expression; therefore, the comparison of metabolite levels in transgenic and control samples separately at different stage of ripeness is desirable.

The statistically significant differences in metabolite levels between 556AZ control and 2AS-AS transgenic fruit were calculated separately for each stage of ripening using ANOVA. Metabolites that were significantly different (*p*-level < 0.05) between fruit of 556AZ and 2AS-AS lines are marked by letter “a” (**Figures 2A–C**). ANOVA shows the difference between two lines to be maximal for pink (P) stage of ripeness (14 metabolites out of 30), followed by breaker (B) and green (G) stages with seven metabolites showing different level in both groups. Only Ala was at a different level in the red tomatoes of the two lines, more in the 556AZ control fruit than that in the 2AS-AS line. In total, the content of 21 metabolites out of 30 is influenced by the transgene expression and this influence is ripening-specific: only Trp and GABA show the different level in two lines throughout all the intermediate stages of ripening, whereas the difference in the content of the other 19 metabolites is observable at one or two stages of ripening only.

The ripening-induced metabolic changes are clearly seen for Ile, Val, Thr, Ala, Tyr, Phe, GABA, myo-inositol, and malate (gradual decrease from green to red tomatoes) and for Glu, Adenosine, AMP, Nucl1, and Nucl2 (gradual increment during ripening).

The influence of transgene expression on ripening process is clearly observable for Thr, Asp, Phe, and myo-inositol; Thr, Phe, and myo-inositol levels being constant in 556AZ control fruit (with a decrease in Phe level at red stage), whereas their levels gradually decrease during ripening of fruit from the transgenic line. Asp level slightly increases with ripening of transgenic fruit but in the 556AZ control fruit the increase is dramatic **Figures 2A,B**).

### INTROGRESSION OF ETHYLENE-DEFICIENT 2AS-AS TOMATO LINE WITH HIGHER-POLYAMINE ACCUMULATING (579HO) TRANSGENIC LINE HIGHLIGHTS DIFFERENTIAL EFFECTS ON METABOLITE PROFILES

**FIGURE 4:**
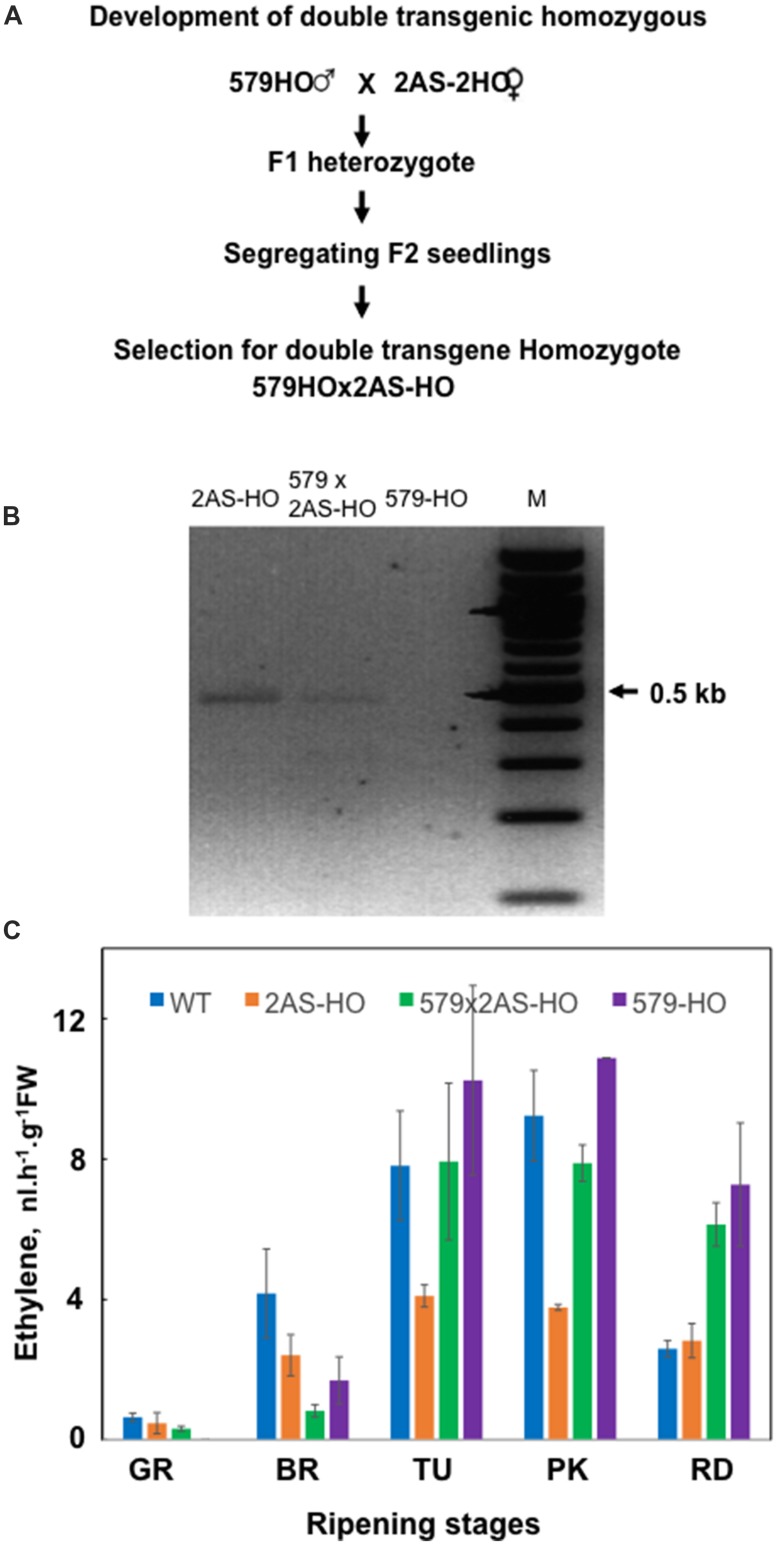
**Generation and characterization of transgenic ACS2-AS and development of double transgenic tomato line expressing both CaMV35S-ACS2-AS and E8:ySAMDC transgenes. (A)** F1 line was developed by pollinating 2AS-AS (2AS-HO) flower with pollen from 579HO line. Plants from the resulting heterozygous seed were characterized. Those that showed presence of both transgenes by RT-PCR confirmed the heterozygote and then their seeds were collected. Fifty segregating seeds from F1 line were grown to maturity and from each segregating line seeds were collected individually. About 20 seedlings from each segregated seed were evaluated for the presence of both transgenes to identify homozygous lines. **(B)** RT-PCR of RNA from 2AS-AS (labeled 2AS-OH) and double transgene homozygous 2AS-AS × 579HO (labeled 579 × 2AS-HO) showing the expression of ACS2-AS transcript. Shown is the 0.5 kb PCR fragment from ACS2 gene. The line 579HO does not harbor ACS2-AS transcript and is therefore negative for its RNA. **(C)** Rate of ethylene evolution by fruits of wild type (WT), 2AS-AS antisense line (2AS-OH), 2AS-AS × 579HO (579 × 2AS-HO) and 579-HO at various stages of ripening. Each time point represents the means + SE from a minimum of three fruits except for the pink (PK) fruits from 579HO line where only two fruits were analyzed.

In order to test which metabolite patterns in the ethylene-deficient tomato fruit are changed by adding to that line the trait of higher accumulation of polyamines, SPD and SPM (Mattoo et al., 2006), we developed a double transgenic tomato line, 2AS-AS × 579HO, by crossing previously developed SPD (and SPM)-accumulating 579HO line (Mehta et al., 2002) and the ethylene-deficient 2AS-AS line as summarized in **Figure 4** (see also Materials and Methods). Surprisingly, this intervention not only reversed the ethylene deficiency in the sibling (the double transgenic line, 2AS-AS × 579HO, in fact, the ethylene levels were significantly higher (**Figure 4C**).

Previously, metabolite profiling of the higher polyamines-accumulating line, 579HO, produced a unique metabolite signature vis a vis the WT or azygous 556 control line, highlighting effects on the aspartate family of amino acid pathway (Mattoo et al., 2006). A similar signature was found in the fruit of the double transgenic line, 2AS-AS × 579HO (**Figure 2A**). Thus, fruit from 2AS-AS × 579HO line had higher levels of Asn (all ripening stages), Gln (G and P stages), and Glu (P and R stages) and lower levels of Asp, GABA (G and B stages), and Trp (from B to R stages) in comparison to fruit from ethylene-deficient 2AS-AS line (**Figure 2A**). The profiles of amino acids Ile, Val, Thr, Ala, Tyr, His, and Phe during ripening remained similar in 2AS-AS × 579HO line with respect to 2AS-AS line fruit. Significant differences (*p*-level < 0.05) between 2AS-AS × 579HO and 2AS-AS lines are marked in **Figures 2A–C** by letter “c.”

Similarly, the profiles of organic acids and sugars were compared between the fruit from the 2AS-AS × 579HO line and the ethylene-deficient 2AS-AS line (**Figure 2B**). The level of citrate was lower and that of fumarate higher in 2AS-AS × 579HO line relative to 2AS-AS line at all the intermediate stages of ripening. Malate level was similar in both lines except for pink (P) stage where its level was about five times lower in 2AS-AS × 579HO fruits. Succinate level was higher in 2AS-AS × 579HO line in comparison to 2AS-AS line at green (G) and pink (P) stages of ripening (**Figure 2B**). At all the ripening stages, myo-inositol level was lower and choline level higher in the 2AS-AS × 579HO line relative to 2AS-AS line.

Other trends noticed in heteroaromatic compounds, adenosine, AMP, ATP + ADP, Nucl1, Nucl2, and trigonelline are presented in **Figure 2C**. Noticeable trends that were not all found to be statistically significant, because of the noise in the data, included higher adenosine (pink, P, stage), AMP (ripe, R, stage), ATP + ADP (lower at pink, P, and significant drop; elevated at ripe, R), Nucl1 and Nucl2 (higher throughout ripening) and trigonelline (decreasing trend at pink, P, and red, R, stages) in the double transgenic line, 2AS-AS × 579HO, relative to the ethylene-deficient 2AS-AS line (**Figure 2C**).

## DISCUSSION

Shelf life of ripening climacteric fruits has been achieved by silencing key gene(s) in ethylene biosynthesis, perception, and signal transduction (Fluhr and Mattoo, 1996). However, whether the altered ethylene production and/or perception affects fruit metabolome or how the resulting fruit quality is achieved is not yet clearly understood. In particular, the levels of a large number of amino acids are significantly reduced in the ACC synthase-impaired tomato fruit, a reduction being more drastic than previously recognized through studies on apple fruit silenced for the expression of ACC oxidase gene (Defilippi et al., 2005). A number of these involve aromatic amino acids (Tyr, Phe, Ile, Val), aspartate family of amino acids (Asp, Asn, Thr, Gln, GABA) and energy/salvage pathway metabolites (adenosine, ATP, ADP), many among which are synthesized from substrates generated from phosphoenolpyruvate and pyruvate, tricarboxylic acid (TCA) cycle intermediates, and amination (Heldt, 2005). Other metabolites affected in ethylene-deficient tomato involve alanine, myo-inositol, and TCA cycle intermediates – citrate, succinate, and fumarate. Gln and Ile were among the metabolites similarly affected in the apple fruit silenced for the expression of ACC oxidase (Defilippi et al., 2005). The depletion of the TCA cycle intermediates in ethylene-deficient tomatoes is likely a reflection on the diminished respiration (Saltveit, 1999). Thus, multiple biochemical pathways are targeted by ethylene in tomato fruit. The synthesis (and dynamics) of these important small molecules occurs in different subcellular compartments in a plant cell, indicating a wide range of ethylene influence on the fruit metabolome (Mattoo et al., 2006; Osorio et al., 2013). Interestingly, the observation that Ile, a major precursor of aroma volatiles, is found commonly affected in relation to ethylene deficiency (Bauchot et al., 1998; Flores et al., 2002; Defilippi et al., 2005) suggests that most ethylene-deficient fruit would be impacted in flavor. However, Ile levels were found to recover to a significant level when 21-day post-harvest apple fruit were exposed to a dose of exogenous ethylene (Defilippi et al., 2005), identifying a critical link between Ile, aroma, and ethylene.

We have previously determined polyamine-responsive metabolome of tomato by analyzing metabolite profiles of the fruit engineered for the expression of heterologous yeast SAMdc gene and fruit-specific accumulation of higher polyamines, SPD, and SPM and comparing these with azygous control fruit (Mattoo et al., 2006). A comparison of metabolites impacted by polyamine addition to ethylene-deficient fruit versus ethylene-deficiency provides a window into the metabolites that these two antagonistic growth regulators (Fluhr and Mattoo, 1996) commonly target but not necessarily in the same direction. These are: Asp, Asn, Gln, Val, citrate, and fumarate. Those metabolites that were suppressed in ethylene-deficient tomato fruit but were neutral to the polyamine response include Thr, Ile, GABA, Ala, and Phe. In this regard, it was interesting to find out which changes in the metabolome of ethylene-deficient tomato fruit would occur when fruit also harbor the high polyamine trait as in the double transgenic tomato developed and presented here. In other words, which polyamine-specific metabolites would dominate and which would behave differently in the double transgenic tomato. Thus, the double transgenic tomato behaved similar to one of its parental, high-polyamine genetic line as regards the following metabolites: Asp, Asn, Glu, Gln, citrate, choline, and ethylene. However, the following metabolites seem to have become a target of the interaction of the two transgenes harbored in the double transgenic fruit: Trp, GABA, succinate, and malate.

Ripening fruits of Ohio 8245 showed increases in the levels of Glu, Asp, His, and Trp but decreased in the levels of GABA, Ile, Val, Gln, Asn, Tyr, and Phe (**Figures 2A–B**). Some of these patterns are similar while others are in contrast with the previously published ripening fruit metabolome of Moneymaker and Ailsa Craig cultivars (Carrari et al., 2006; Osorio et al., 2011). We observed increases in the levels of Glu and Asp but not of Phe, Ile, and Ala as reported in Moneymaker and Ailsa Craig cultivars. Phe Ile and Ala declined during the fruit ripening of Ohio 8245 cultivar (**Figure 2A**). Levels of GABA and Val declined in all three investigations (present study; Carrari et al., 2006; Osorio et al., 2011), but the levels of Thr, Gln, Asn, His, and Trp did not show significant changes during ripening of fruit from Ohio8245 line. Unlike the *Nr* mutant fruit, which exhibited decrease in Thr, fruit from 2AS-AS exhibited ripening-associated increase in this amino acid. Fruits from the 2AS-AS line showed decline in Val, Ala, Glu Gln Asp, Asn, Phe, and GABA during ripening, implying that even 50% reduction in ethylene production has a major effect on fruit metabolome. It is possible that different threshold levels of ethylene may regulate particular events/processes in a fruit. However, decline in β-Ala and GABA in *rin*, *Nor*, and 2AS-AS (ethylene biosynthesis attenuated) mutants indicates that ethylene regulates their accumulation during fruit ripening.

Among the organic acids, the levels of malate and fumarate declined noticeably in 2AS-AS, but increased several-fold in the double transgenic fruits, likely reflecting diminished respiration in 2AS-AS fruit in contrast to higher respiration in double transgenic fruit. We have previously shown that 579HO fruit exhibit several-fold higher respiration during ripening as compared to the WT and 556AZ fruit (Mattoo et al., 2006). These results are different from those reported in Moneymaker and Ailsa Craig where an increase in succinate and a decrease in malate was evident (Carrari et al., 2006; Osorio et al., 2011). Also, unlike ripening mutant(s) in which malate and succinate do not change during fruit ripening (Osorio et al., 2011), reduction in ethylene production significantly affected levels of citrate, succinate, and malate (**Figure 2B**), the data that are in line with ethylene regulation of respiration (Saltveit, 1999), largely involving the TCA cycle intermediates (Mattoo et al., 2006).

Levels of glucose and fructose remained similar throughout the fruit ripening of control fruit with a significant increase in sucrose levels only at the onset of ripening (breaker stage; **Figure 2B**). Contrasting results have been reported for changes in sugar levels in Moneymaker and Ailsa Craig fruits. Whereas a linear increase in glucose and fructose levels was reported in Moneymaker fruit, such changes were not seen in Ailsa Craig fruit (Carrari et al., 2006; Osorio et al., 2011). Such changes in 2AS-AS were not apparent in the present study, indicating a more complex role of ethylene/ripening associated with sugar metabolism. Reduction in glucose, fructose, citric, and malic acids has been reported in strawberry line with altered ethylene perception due to the overexpression of *etr1-1* ethylene receptor mutant (Merchante et al., 2013).

The ingression of SAMdc overexpression in 2AS-AS × 579HO cross resulted in partial recovery of several metabolites otherwise affected by reduction in ethylene in the 2AS-AS line (**Figures 2A–C**). The metabolic parameters that exhibit this partial to full recovery include Ile, Glu, Gln, Asn, succinate, fumarate, formate, compound B, Nucl1, and Nucl2. This recovery is associated with 2 to 3-fold higher ethylene production in 556HO because of SAMdc overexpression (Mehta et al., 2002; see also **Figure 4C**). The enhanced metabolic activity due to higher polyamines, SPD, and SPM, is associated with increased levels of Ile, Val, Thr, Asn, His, Trp, succinate, fumarate, formate, compound B, choline, adenosine, Nucl1, and Nucl2, as reported previously (Mattoo et al., 2006, 2007; Srivastava et al., 2007). Thus, polyamines SPD and SPM prominently alter the metabolic changes associated with the double transgenic tomato fruit. These findings are in line with the determined metabolome previously reported and discussed in relation to the genetic event of introducing ySAMdc transgene in tomato (Mattoo and Handa, 2008; Handa and Mattoo, 2010; Mattoo et al., 2010).

This study provides new dimension in light of other reports in the literature (and some mentioned above) showing that suppression of ethylene has measurable impact on the metabolome and aroma of a commodity. Thus, altering ethylene biosynthesis or signaling in plants, be that by a chemical treatment or using genetic means (mutants or transgenes), to prolong shelf life of fruits limits the metabolic potential (nutrition) of such fruit, which needs to be ascertained and then rectified/salvaged by additional treatments including applying exogenous ethylene or creating a double transgenic event as shown here.

## Conflict of Interest Statement

The authors declare that the research was conducted in the absence of any commercial or financial relationships that could be construed as a potential conflict of interest.
